# Ethanol responsive lnc171 promotes migration and invasion of HCC cells via mir-873-5p/ZEB1 axis

**DOI:** 10.1186/s12885-024-12309-3

**Published:** 2024-05-01

**Authors:** Shiping Huang, Zhouxiang Liao, Xiao He, Zhenyu Song, Xi Fang, Sha Wen, Lichao Yang, Hui Li, Qi Zhang, Wanling Mo, Xiaojing Cheng, Min He, Xuejing Huang

**Affiliations:** 1 School of Public Health, Guangxi Medical University, Nanning, 530021 China; 2 School of Public Health, Guilin Medical School, Guilin, 541199 China; 3 Laboratory Animal Center of Guangxi Medical University, Nanning, 530021 China; 4 Life Sciences Institute of Guangxi Medical University, Nanning, 530021 China; 5 Key Laboratory of High-Incidence-Tumor Prevention & Treatment, Guangxi Medical University, Ministry of Education, Nanning, 530021 China

**Keywords:** Hepatocellular carcinoma, lncRNA, ceRNA, Ethanol, Migration, Invasion

## Abstract

**Backgrounds:**

Long nonconding RNAs (lncRNAs) have been found to be a vital regulatory factor in the development process of human cancer, and could regarded as diagnostic or prognostic biomarkers for human cancers. Here, we aim to confirm the expression and molecular mechanism of RP11-171K16.5 (lnc171) in hepatocellular carcinoma (HCC).

**Methods:**

Screening of differentially expressed lncRNAs by RNA sequencing. Expression level of gene was studied by quantitative real-time PCR (qRT-PCR). The effects of lnc171, mir-873-5p, and ethanol on migration and invasion activity of cells were studied used transwell assay, and luciferase reporter assay was used to confirm the binding site.

**Results:**

RNA sequencing showed that **l**nc171 was markedly up-regulated in HCC. siRNA-mediated knockdown of lnc171 repressed the migration and invasion ability of HCC cells. Bioinformatic analysis, dual luciferase reporter assay, and qRT-PCR indicated that lnc171 interacted with mir-873-5p in HCC cells, and Zin-finger E-box binding homeobox (ZEB1) was a downstream target gene of mir-873-5p. In addition, lnc171 could enhance migration and invasion ability of HCC cells by up-regulating ZEB1 via sponging mir-873-5p. More interestingly, ethanol stimulation could up-regulate the increase of lnc171, thereby regulating the expression of competing endogenous RNA (ceRNA) network factors which lnc171 participated in HCC cells.

**Conclusions:**

Our date demonstrates that lnc171 was a responsive factor of ethanol, and plays a vital role in development of HCC via binding of mir-873-5p.

**Supplementary Information:**

The online version contains supplementary material available at 10.1186/s12885-024-12309-3.

## Backgroud

Hepatocellular carcinoma (HCC) is the fourth most common type of cancer and the second most common cause of cancer associated deaths [1]. There are nearly one million new cases of HCC and over ¾ million HCC related deaths worldwide every year [2, 3]. Unfortunately, although progress have been made in early diagnosis and specialized treatments in recent years, only limited HCC patients get help, and majority of HCC patients still have bad prognosis [4, 5]. Therefore, understanding the pathological conditions underlying carcinogenesis and development of HCC, seeking new diagnosis and therapeutic method, is of great significance for ameliorating the quality of life and reducing adverse prognosis in HCC patients.

Long noncoding RNAs (lncRNAs) are members of the non-coding transcriptome family with a length of over 200 nucleotides and play significant functional roles in multiple human cancer, including HCC [5–7]. Current research illustrated that lncRNA could conduct as competing endogenous RNA (ceRNA) and take part in regulating numerous cellular processes [8]. ceRNA network is a novel mechanism that reveals RNA (lncRNA-miRNA-mRNA) interactions [9] and it uncover that cytoplasm lncRNAs can regulate target genes by competing with mRNA for microRNA (miRNA) binding site [5, 6, 10]. Zhang et al. found that over-expression of NEAT1 combine with mir-362-3p, indirectly enhance the MIOX expression, lead to promote erastin and RSL3 induced ferroptosis in HCC cells [6].

Dysregulation of lncRNA expression were commonly seen in numerous cancer [11–13]. However, what factors may cause changes of lncRNA expression in human body? Several studies have shown that many pathogenic factors or disease inducements may lead to abnormal expression of lncRNA [14, 15]. For example, alcohol, one of the risk factors of HCC, that could induce alcoholic hepatitis which gradually develop into alcoholic cirrhosis and eventually leading to the occurrence of HCC [16]. And alcohol have been shown to be an epigenetic modulator that could stimulate cells to secreted variety of cytokine factors including lncRNA [17–19]. However, the function and mechanism of lncRNA in the process of alcohol-induce liver injury and HCC remains unknow.

In the present study, we found a new function of lncRNA, RP11-171K16.5 (lnc171), was aberrantly upregulated in human HCC. Furthermore, we confirmed that lnc171 could competitively sponge on mir-873-5p to affect its downstream gene-ZEB1 resulting to enhance HCC cells migration and invasion. More interestingly, we found that lnc171 was a responsive factor of ethanol and determined the regulatory mechanism involved in ethanol-induced lnc171 in HCC cells.

## Materials and methods

### Patients blood sample and RNA-Sequencing analysis

The blood samples of HCC patients and healthy controls (HC) were obtained for RNA sequencing were all from the First Affiliated Hospital of Guangxi Medical University. The methods of blood collection and RNA-sequencing refer to the previous published articles [20]. Briefly, RNA of Plasma exosomes from HCC patients and healthy controls were extracted for RNA-sequencing. The raw read sequences were filtered to remove adapter sequences and low-quality reads (more than 20% bases with quality less than 20, or reads with more than 10% undefined nucleotides) by using Skewer and the FastX-Toolkit software. Reads were mapped with human reference rRNA sequences (GenBank and GENCODE v26); reads that did not map with rRNA (clean reads) were subjected to subsequent analysis. Clean reads were aligned to human reference genome assembly (GRCh38) to define mRNA and lncRNA profiles by HISAT2 aligner with the default parameters. Clean reads were also annotated to GENCODE (v26) and the transcript expression level was quantified by the Expectation-Maximization (RSEM) package. Expression levels were calculated by reads per kilobase of transcript per million mapped reads. The sequencing results of HCC patients and healthy controls were compared. When the |fold change|≥2.0 and *P* < 0.05 of the transcript, it could be considered that the expression of the transcript was different between HCC patients and healthy controls.

### Cell culture

Normal hepatocyte (HL7702) and HCC cells (Hep3B and Huh7) were obtained from Chinese Academy of Sciences (Shanghai, China). Dulbecco’s modified Eagle’s medium (Invitrogen, USA) was used to culture HL7702 and Huh7 cells, and Minimum Essential medium (Invitrogen, USA) was used to culture Hep3B. The composition of the cell culture medium was as follows: 98% culture medium, 10% fetal bovine serum (Thermo Fisher Scientific, USA), and 1% penicillin-streptomycin (Thermo Fisher Scientific, USA). All cells were placed in a 37℃ humidified incubator with 95% air and 5% CO_2_.

### Establishment of ethanol treated cell model

HL7702 (2.5 × 10^5^ per well), Huh7 (2.5 × 10^5^ per well) and Hep3B (3.0 × 10^5^ per well) cells were plated in 6-well plates with 2 ml complete medium for 12 h, then remove the culture medium and added 2 ml of 200 mM ethanol (24 µl anhydrous ethanol to 2 ml complete medium) into each well and cultured for 48 h to establish ethanol treated cell model. The control group was added with 2 ml complete medium.

### Cell transfection

Three small interfering RNAs of lnc171, inhibitor and mimics of mir-873-5p were designed and construction by Sangong Biotech (Table S1) (Shanghai, China). siRNA, mir-873-5p inhibitor and mimics were transfected by Lipofectamine 3000 (Thermo Fisher Scientific, USA). Before transfection, cells were plated in 6-well plates (HL7702 and Huh7 cells: 2.5 × 10^5^ per well; Hep3B cells: 3.0 × 10^5^ per well) with 2 ml complete medium for 12 h. Three si171, mir-873-5p mimics, mir-873-5p inhibitor, and negative control (NC) were transfected, respectively, into the appropriate cell group, then added with 2 ml Opti-MEM medium without SBF per well following the manufacturer’s instruction.

### Transwell assay

Transwell chambers (pore size 8 μm; Coning, USA) were used to conduct transwell assay with or without matrigel (BD Biosciences, USA). After transfection or ethanol treatment, cells (HL7702 and HepG2 cells: 5 × 10^4^ per well; Hep3B cells: 7 × 10^4^ per well) were cultured in the dishes with 200 ml serum-free medium. The bottom chamber was added with 600 µl of completed cultured medium. After incubation for 48 h, cells that migrated or invaded to the bottom of the filter membrane were fixed with methanol, stained with 0.5% crystal violet solution then photographed. Three fields were randomly selected and the relative cell number was calculated.

### Quantitative real-time PCR analysis

The process of total RNAs extraction, RNA reverse transcription, and qRT-PCR was according to our previously study [20]. The 2^−ΔΔCt^ method was used to calculate the relative folding changes of gene expression. U6 andβ-actin were used as an endogenous control. Primer sequences were listed in Table S2.

### Western blot assay

The process of obtain the cellular protein, protein quantitation, protein electrophoresis, and protein bands detection were according to our previously study [20]. Briefly, RIPA Buffer (Beyotime Biotechnology, Shanghai, China) was used to lyse cells. The concentrations of protein were quantified using BCA kit (Beyotime Biotechnology, Shanghai, China). Then separated the protein by sodium dodecyl sulfate (SDS)-PAGE on 10% gels and transferred to poly-vinylidene difluoride (PVDF) membranes (the membranes were cut to appropriate size prior to hybridisation with antibodies during blotting) (Sigma-Alcdrih, St. Louis, MO, USA). After overnight incubation with first antibodies, then incubated for 2 h in the presence of secondary antibody and washed 3 times with TBST for 5 min. Quantity-one software (Bio-Rad Laboratories, USA) using the ECL-chemiluminescent kit. The signal of protein bands was quantified by ImageJ software for Windows (NIH, USA). The first antibody was ZEB1 (200 kDa, ab203829, dilution 1:500, Abcam, UK), GAPDH (37 kDa, 14C10, dilution 1:1000, Cell Signaling Technology, USA). The secondary antibody was HRP-conjugated Goat anti-rabbit IgG (abs20040ss, dilution 1:1000, absin, China).

### Luciferase reporter assay

The binding sites between lnc171 and mir-873-5p, and between mir-873-5p and ZEB1 were predicted by online website. The DNA fragments of wild-type-lnc171 (lnc171-WT), mutant-lnc171 (lnc171-MUT), Wild-type-ZEB1, and mutant-ZEB1 were cloned into the pmiRGLO vector (Promega, USA). The HEK-293T cells was selected to perform luciferase reporter assay and were seeded on 96-well plate, then mir-873-5p NC or mir-873-5p mimics were transfected with wild- or mutant-type luciferase plasmids using Lipofectamine 3000 for 48 h. After transfection, the fluorescence intensity of firefly and Renilla was measure by Luciferase reporter kit (Yisheng Bio-Technology, China) and Multifunctional enzyme labeling instrument (Tecan, CH).

### Bioinformatics analysis

The target gene of lnc171 or mir-873-5p were predicted by miRDB (https://mirdb.org/), TargetScan (https://www.targetscan.org/), and MicroT-CDS (https://dianalab.e-ce.uth.gr/html/dianauniverse/index.php?r=microT_CDS). miRDB could show the binding sites of target genes. UALCAN (https://ualcan.path.uab.edu/index.html) was used to obtain expression level data in HCC patients of ZEB1 that link TCGA database. ONCOLNC (http://www.oncolnc.org/) was used to obtain survival data of ZEB1 which link TCGA database.

### Statistical analysis

All data were analyzed using SPSS20.0 software. Using GraphPad Prism 5.0 software to draw graphs. All data were represented as the mean ± S.D. The comparison of the data between two experimental groups was conducted by using Student’s t-test, and the comparison of multiple groups was conducted by using one-way ANOVA. *p* < 0.05 (*), *p* < 0.005 (**) and *p* < 0.0005 (***), were significant statistical.

## Results

### Lnc171 was highly expressed in HCC, and ethanol consumption enhance lnc171 expression

**Fig. 1:**
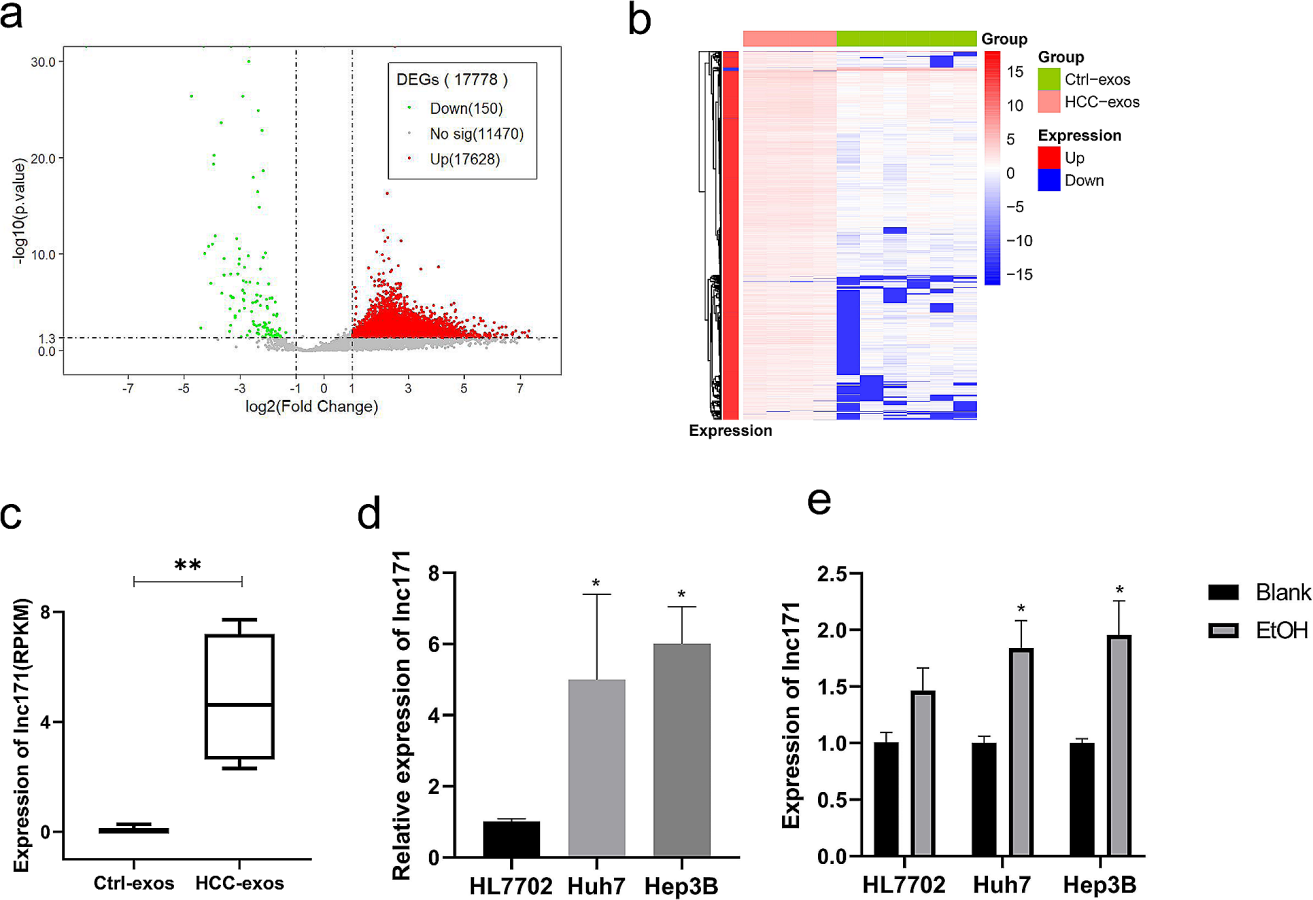
lnc171 was over-expressed in HCC and ethanol stimulation enhance this phenomenon. (**a**) Volcano plot showing DE-lncRNAs between HCC-exos and healthy Ctrl-exos. (**b**) Hierarchical clustering of DE-lncRNA between HCC-exos and Ctrl-exos. (**c**) Expression level of lnc171 between HCC-exos and Ctrl-exos by RNA-Sequence. (**d**) Respective expression level of lnc171 in HCC cells was detected by qRT-PCR. (**e**) Respective expression level of lnc171 in hepatocyte and HCC cells after stimulated by ethanol was detected by qRT-PCR. *p < 0.05, **p < 0.005

According to the fold change ≥ 2.0 and *p* < 0.05, we found a total of 17,778 DE-lncRNAs from the result of RNA-sequence (Fig. 1a, b), and lnc171 was highly up-regulated in plasma exosome of HCC patients (*p* < 0.05) (Fig. 1c). Then we used qRT-PCR to measure the level of lnc171, and found that lnc171 was higher expressed in three HCC cell lines than in HL7702 cells (*p* < 0.05) (Fig. 1d). Some studies have reported that ethanol could induce changes of lncRNA levels in cells [17–19], so we constructed a cell model of ethanol treatment to observe whether ethanol can cause changes of lnc171 levels in HCC cells. Before establishing the cell model, we treated cells with gradient concentration of ethanol and measured cellular activity at 24 h, 48 h, and 72 h after ethanol treatment. The results showed that cells still maintained good activity when treated with 200 µM ethanol for 48 h (*p* > 0.05) (Figure S1). Hence, we chose to treat cells with 200 µM ethanol for 48 h to construct the cell model. Then we found that after ethanol stimulation, the expression of lnc171 was higher than before stimulation in HCC cells (*p* < 0.05) (Fig. 1e).

### Ethanol promoted the migration and invasion of HCC cells by inducing an increase of lnc171

**Fig. 2:**
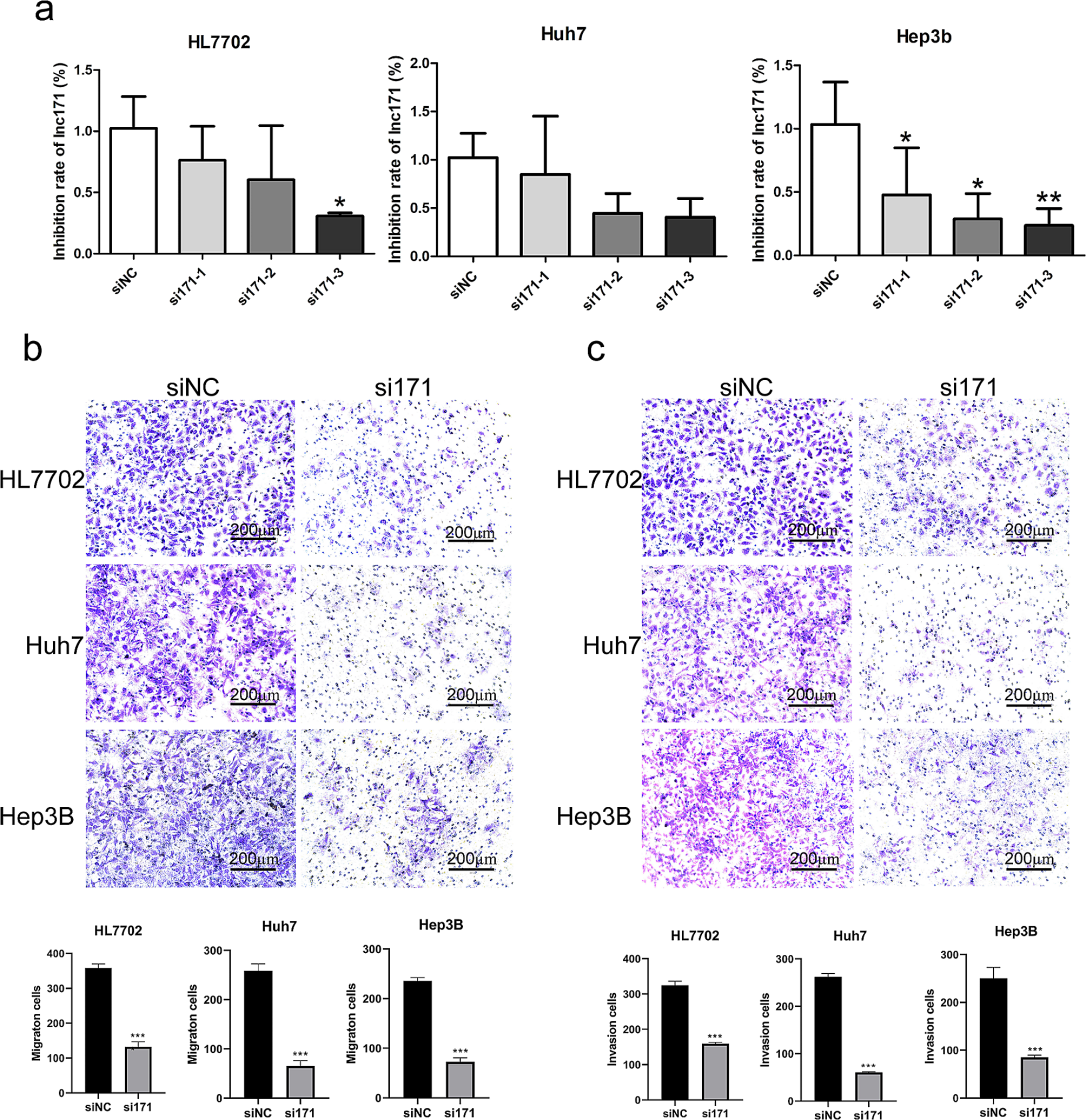
Knockdown of lnc171 significantly inhibit cell migration and invasion phenotypes in hepatocyte and HCC cells. (**a**) Inhibitory rate of lnc171 after transfection with three siRNA respectively detected by qRT-PCR. Cell migration (**b**) and invasion (**c**) abilities were indicated by transwell assays (20X). *p < 0.05, **p < 0.005, ***p < 0.0005

To explore the impact of lnc171 on the phenotype on cells, we knocked down lnc171 in HCC cells by using three siRNAs. As shown in Fig. 2a, lnc171 was reduced in cells when transfected with three siRNA, and the inhibition effect of si171-3 was the most obvious in HL7702 (*p* < 0.05) and Hep3B (*p* < 0.005), therefore, si171-3 was used for cell transfection in subsequent experiments and labeled as si171. After transfection with si171, we found that silencing lnc171 significantly reduced migration and invasion in HCC cells (*p* < 0.0005) (Fig. 2b, c).

**Fig. 3:**
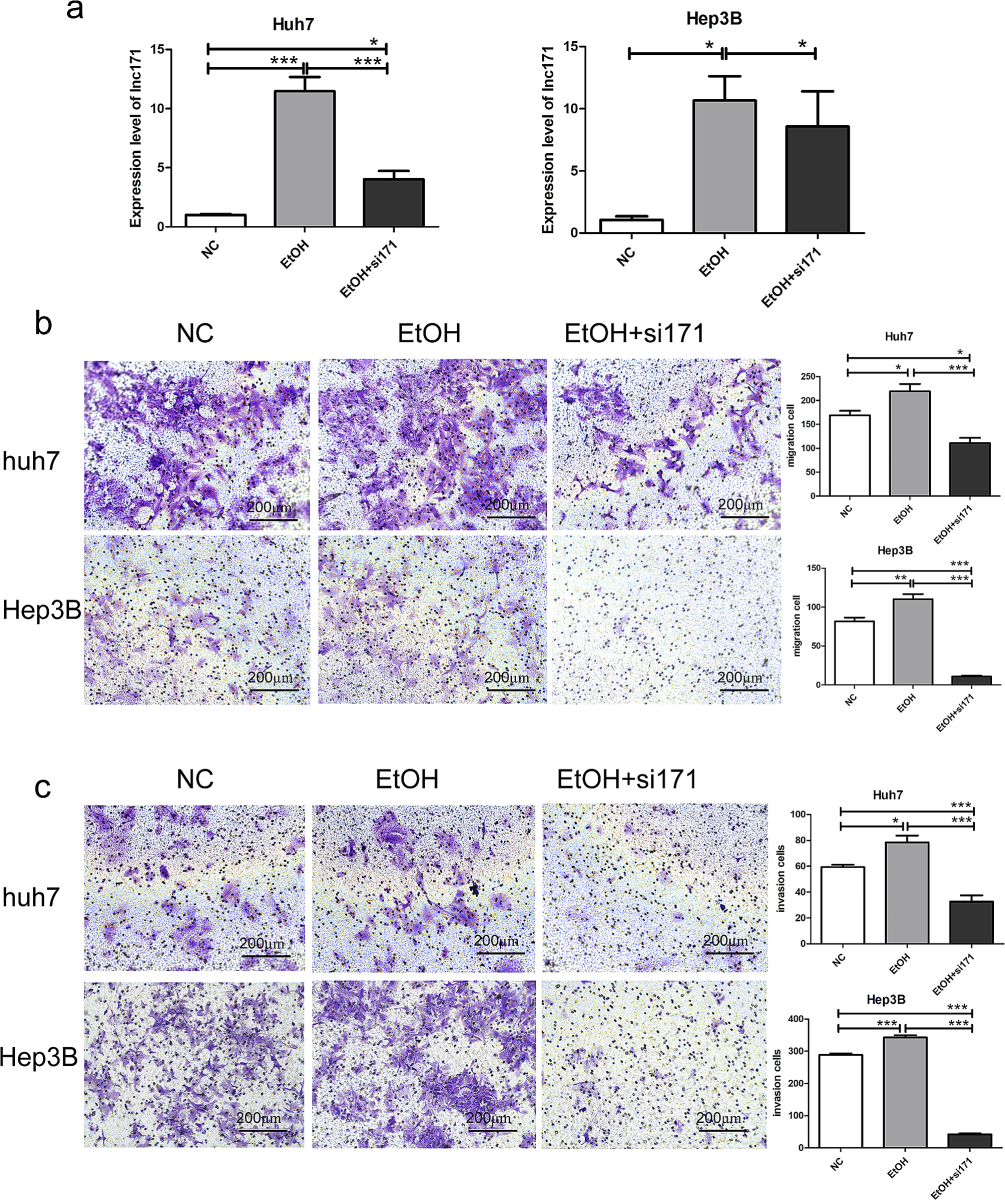
Ethanol stimulation up-regulate the expression of lnc171 and promote the migration and invasion of HCC cells. (**a**) Respective expression level of lnc171 in HCC cells after ethanol stimulated or ethanol stimulated combined with siRNA transfection was detected by qRT-PCR. Cell migration (**b**) and invasion (**c**) abilities were indicated by transwell assays (20X). *p < 0.05, **p < 0.005, ***p < 0.0005

At previous experiments, we observed that lnc171 was significantly increased in HCC cells when stimulated by ethanol. In order to further demonstrate that lnc171 was an ethanol reactive factor and to understand the role of ethanol in cell migration and invasion, we silenced the expression of lnc171 after ethanol stimulation to test the changes in migration and invasion in HCC cells. The results showed that when irritated by ethanol, the expression of lnc171 was significantly up-regulated in Huh7 and Hep3B cells (*p* < 0.05) (Fig. 3a), and the migration and invasion of these two cells were significantly enhanced (*p* < 0.05) (Fig. 3b, c); however, when si171 was transfected after ethanol stimulation, the expression of lnc171 was clearly down-regulated (*p* < 0.05) (Fig. 3a), and the migration and invasion ability of Huh7 and Hep3B cells were also evidently decreased (*P* < 0.05) (Fig. 3b, c).

### Lnc171 is a sponge of mir-873-5p

**Fig. 4:**
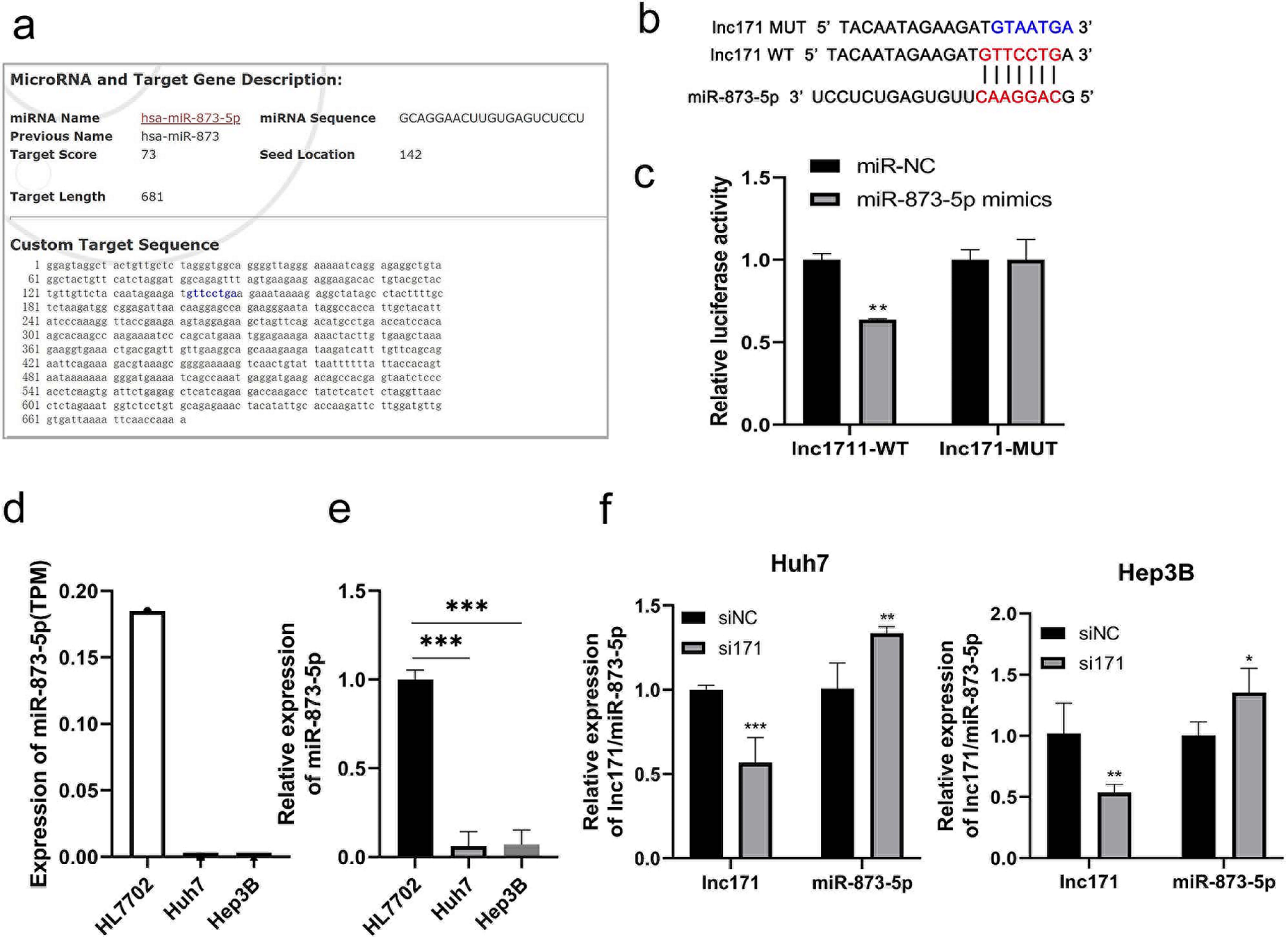
Targeting relationship between lnc171 and mir-873-5p. (**a**) mir-873-5p putative binding site on lnc171 by miRDB. (**b**) Construction schematic diagram of lnc171-wild-type (lnc171-WT) and lnc171-mutant (lnc171-MUT) luciferase reporter vector. (**c**) Relative luciferase activity was detected in 293T cells cotransfected with lnc171-WT or lnc171-MUT vectors and mir-873-5p mimic or miR-negative control (miR-NC). (**d**) Expression level of mir-873-5p between HCC-exos and Ctrl-exos by RNA-Sequence. (**e**) Expression level of mir-873-5p in hepatocyte and HCC cells was detected by qRT-PCR. (**f**) Expression level of lnc171 and mir-873-5p in HCC cells after transfection with siRNA was detected by qRT-PCR. *p < 0.05, **p < 0.005, ***p < 0.0005

LncRNAs were known could sever as ceRNA to regulate miRNAs’ expression and biological activity [8]. Whether lnc171 could also act as ceRNA to participate in the migration and invasion process of HCC cells? In order to figure out the answer, we used online prediction software miRDB to predict the potential targets of lnc171. Then we found lnc171’s 3’-UTR ends include a binding site for mir-873-5p (Fig. 4a). And the result of dual-luciferase reporter assay also indicated that mir-873-5p mimic could remarkably suppressed the activity of luciferase of lnc171-WT 3’-UTR in 293T cells, however, no luciferase activity was observed in mir-873-5p mimic on mutant 3’UTR (*p* < 0.005) (Fig. 4b, c). In addition, transcriptome sequencing and qRT-PCR analysis also proved that the expression of mir-873-5p was opposite to lnc171 in Huh7 and Hep3B cells (Fig. 4d, e). Silence of lnc171 would inhibit the levels of lnc171 but up-regulated mir-873-5p levels in Huh7 and Hep3B cells (Fig. 4f). All these results indicating that mir-873-5p might serve as a target of lnc171.

### Mir-873-5p inhibits migration and invasion of HCC cells via targeting ZEB1

**Fig. 5:**
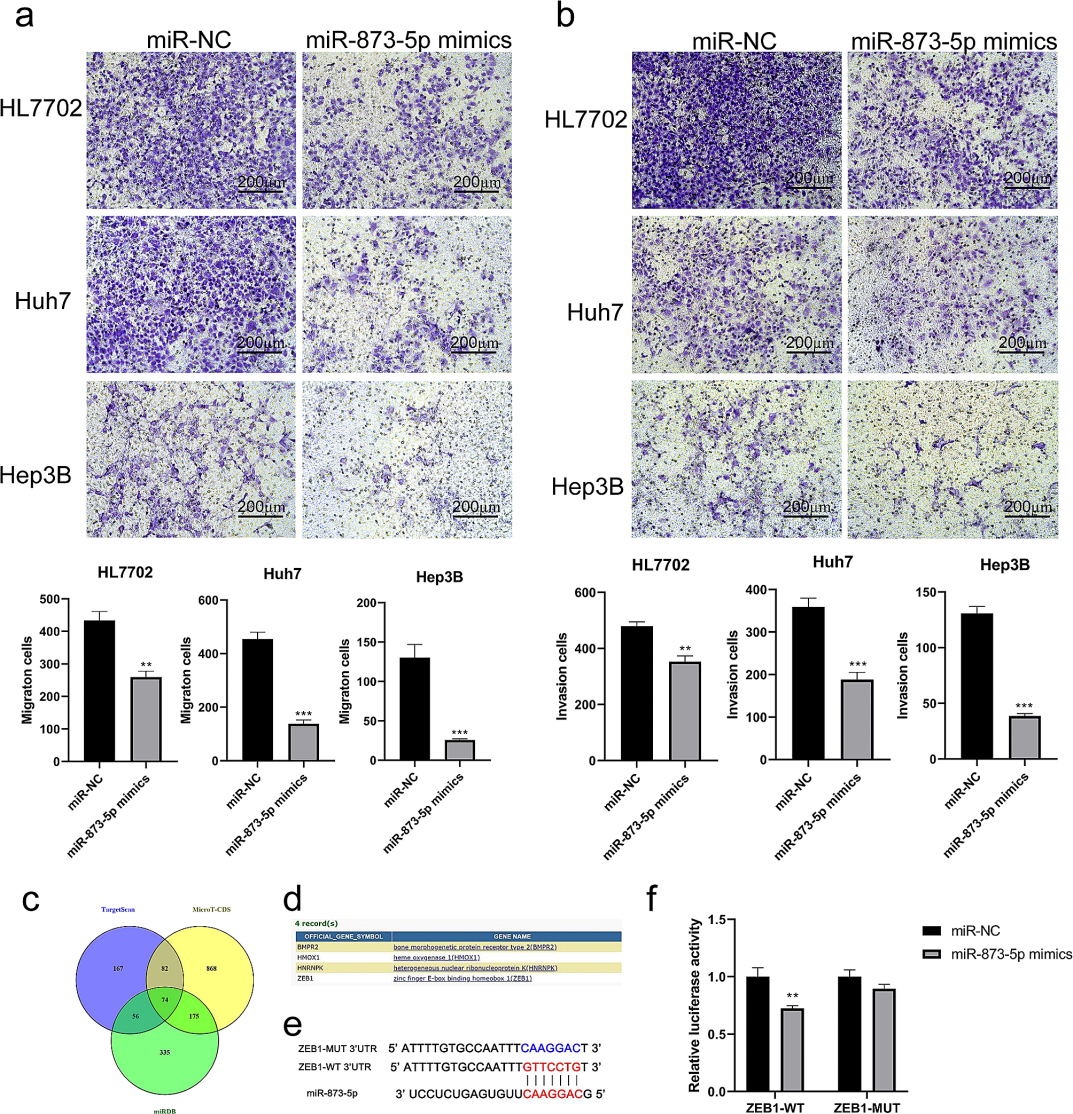
mir-873-5p inhibited cell migration and invasion by ZEB1. Cell migration (**a**) and invasion (**b**) abilities were indicated after transfected with mir-873-5p mimic or miR-NC by transwell assays (20X). (**c**) Venn diagram of target genes of mir-873-5p predicted by three target gene prediction sites, a total of 74 overlapping genes were screened. (**d**) KEGG pathway enrichment of 74 target gene of mir-873-5p. (**e**) Construction schematic diagram of ZEB1-WT and ZEB1-MUT luciferase reporter vector. (**f**) Relative luciferase activity was detected in 293T cells cotransfected with ZEB1-WT or ZEB1-MUT vectors and mir-873-5p mimic or miR-NC. *p < 0.05, **p < 0.005, ***p < 0.0005

We transfected mir-873-5p mimics into HL7702s, Huh7 and Hep3B cells to examine the influence of mir-873-5p in the migration and invasion in HCC cells. The results showed that over-expression of mir-873-5p suppressed the abilities of migration and invasion in HL7702, Huh7 and Hep3B cells compared with miR-NC group (*p* < 0.005) (Fig. 5a, b).

miRNA drive its function by regulating the expression of downstream genes. To identify the main target genes of mir-873-5p, the TargetScan, microT-CDS and miRDB were used. The number of target genes predicted by each database were 379, 1199 and 640, respectively. Then using Venny2.1 to intersect the results of three databases, and 74 intersecting target genes were obtained (Fig. 5c). KEGG pathway enrichment was performed on 74 target genes using DAVID, and four genes, BMPR2, HMOX1, HNRNPK and ZEB1, were enriched on the “microRNAs in cancer” signal pathway (Fig. 5d). Among these four gene, Zinc finger E-box binding homeobox 1 (ZEB1) is a key factor associated with tumor migration and invasion [21]. We used TagetScan Human 7.2 to analysis and find a binding site on 3’-UTR ends of ZEB1 for mir-873-5p (Fig. 5e), and a dual-luciferase reporter gene assay showed that mir-873-5p mimic significantly inhibited the luciferase activity of ZEB1 WT3’UTR in 293T cells, whereas mir-873-5p mimic had no significant effect on the luciferase activity of MUT3’UTR (*p* < 0.005) (Fig. 5f). This founding suggests us that mir-873-5p may regulate cell migration and invasion by ZEB1.

### Lnc171 promotes migration and invasion of HCC cells by mir-873-5p/ZEB1 axis

**Fig. 6:**
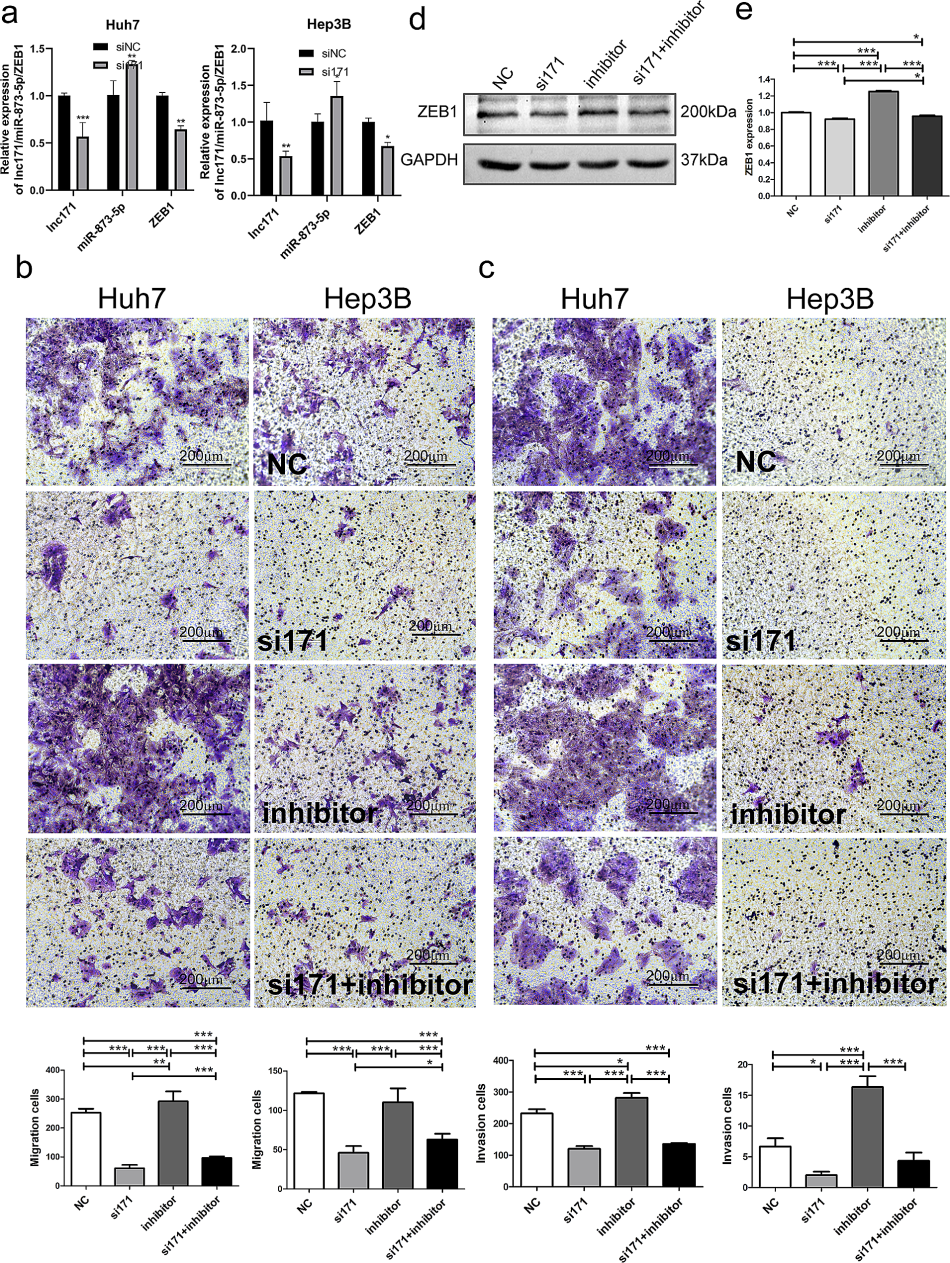
lnc171 enhance cell migration and invasion by targeted mir-873-5p/ZEB1 axis. (**a**) Respective expression level of lnc171/mir-873-5p/ZEB1 in HCC cells after transfected with siRNA was detected by qRT-PCR. Cell migration (**b**) and invasion (**c**) abilities were indicated after transfected with siRNA or mir-873-5p inhibitor or siNC by transwell assays (20X). (**d**) Western blot images show protein expression change of ZEB1 after transfected with siRNA or mir-873-5p inhibitor or siNC. *p < 0.05, **p < 0.005, ***p < 0.0005

As we had confirmed that mir-873-5p was targeted by lnc171, next we want to investigate whether lnc171 could affect the expression of ZEB1 which downstream of mir-873-5p. The results showed that compare with control, the expression level of mir-873-5p was low, while the mRNA and protein level of ZEB1 increased in Huh7 and Hep3B cells before lnc171 was silenced. In contrast, after lnc171 silencing, mir-873-5p was increased, while ZEB1 was decreased in both mRNA and protein level (*p* < 0.05) (Fig. 6a). Therefore, we have demonstrated that lnc171 could up-regulate the level of ZEB1 through inhibit the expression of mir-873-5p. However, does lnc171 regulate the migration and invasion ability of HCC cells through mir-873-5p/ZEB1axis? The results indicated that when lnc171 was inhibited, the migration and invasion ability was damaged in HCC cells; when mir-873-5p was inhibited, the migration and invasion of HCC cells was increased; however, when lnc171 and mir-873-5p were simultaneously inhibited, the migration and invasion ability of HCC cells was remained lower compared to the control (*p* < 0.05) (Fig. 6b, c). In addition, the results of western blot showed that ZEB1 mRNA levels decreased after lnc171 silencing, while increased after mir-873-5p inhibition. When both lnc171 and mir-873-5p were inhibited, ZEB1 protein levels still decreased compared to the control (Fig. 6d, e). These results suggesting that lnc171 could up-regulate the expression of ZEB1 by inhibiting the function of mir-873-5p, thereby promoting the migration and invasion of HCC cells.

### Ethanol up-regulated lnc171 and enhance HCC cells migration and invasion by mir-873-5p/ZEB1 axis

**Fig. 7:**
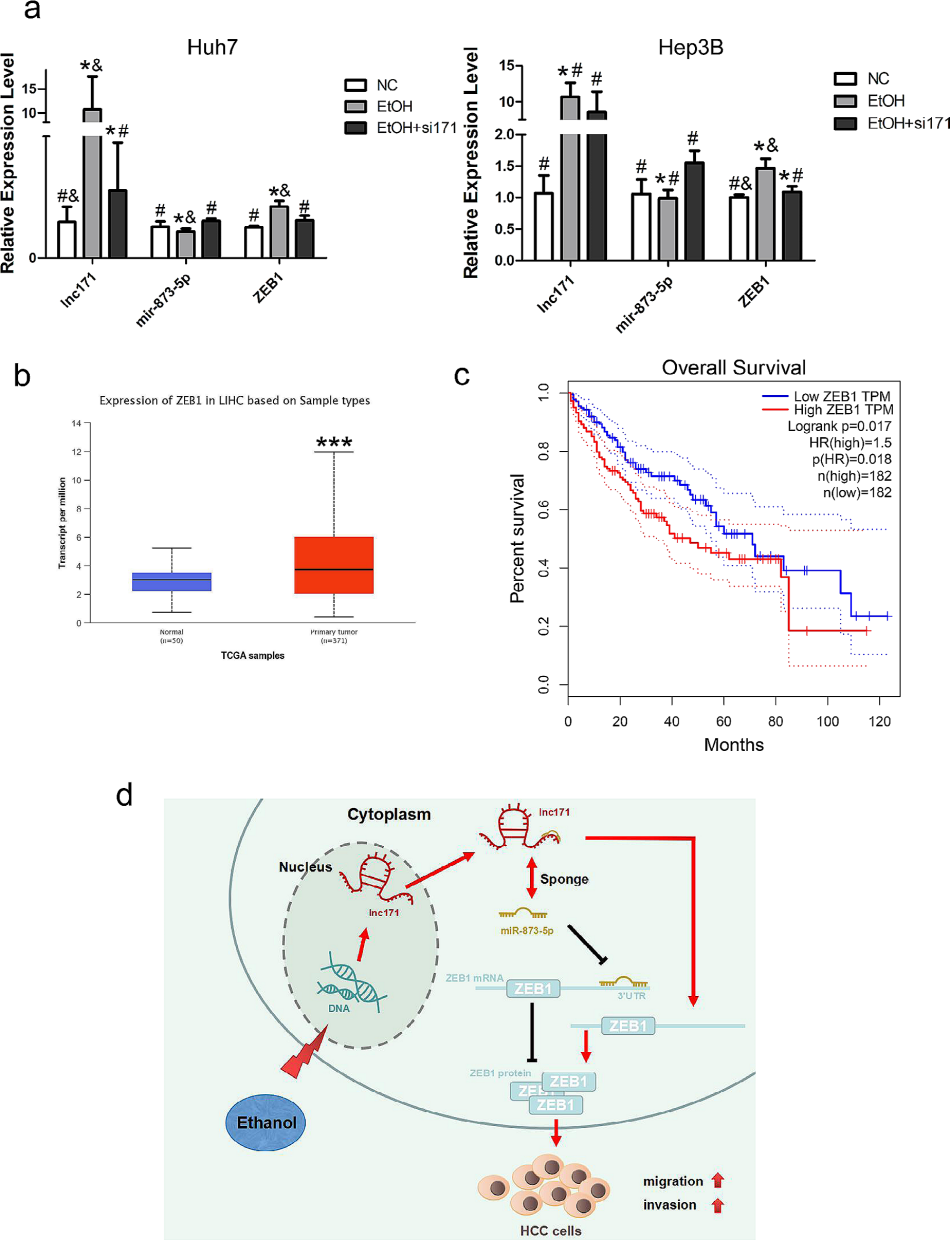
Ethanol stimulation up-regulated the expression of lnc171 and promoted the proliferation of HCC cells through mir-873-5p/ZEB1 axis. (**a**) Respective expression level of lnc171/mir-873-5p/ZEB1 in HCC cells after ethanol stimulated or ethanol stimulated combined with siRNA transfection was detected by qRT-PCR. *p < 0.05 vs. NC, #*p* < 0.05 vs. EtOH, &*p* < 0.05 vs. EtOH + si171. (**b**) Expression of ZEB1 between normal and primary tumors in HCC patients in UALCAN database. (**c**) Overall survival (OS) curves for ZEB1 in low- and high-risk groups in the HCC patients using the Kaplan-Meier method in ONCOLNC database. (**d**) Working model of lnc171/mir-873-5p/ZEB1 axis stimulating by ethanol in HCC. *p < 0.05, **p < 0.005, ***p < 0.0005

Lnc171 proved to be a responsive factor of ethanol in our experiments. We silenced the expression of lnc171 after ethanol stimulation to detect the expression level of mir-873-5p and ZEB1 in HCC cells and found that ethanol irritation could increase the expression level of lnc171 and ZEB1 but decrease the level of mir-873-5p; nevertheless, when si171 was transfected after ethanol stimulation, the expression of lnc171 and ZEB1 was clearly down-regulated but mir-873-5p was up-regulated (*p* < 0.05) (Fig. 7a). In summary, ethanol stimulation can up-regulate the expression level of lnc171, thereby inhibiting the expression level of mir-873-5p and up-regulating the mRNA level of ZEB1. These results suggesting that ethanol can participate in the mir-873-5p/ZEB1 axis by up-regulating the level of lnc171 and promote the migration and invasion of HCC cells (Fig. 7d). ZEB1 is the final target protein of the ceRNA regulatory network (lnc171—mir-873-5p—ZEB1) in which lnc171 involved in, so we used an online database to further explore its value as a biomarker of HCC. The results indicated that the expression of ZEB1 in HCC tissues was significantly higher than that in normal tissues (*p* < 0.0005) (Fig. 7b), and HCC patients whose highly expression of ZEB1 had a poor prognosis (*p* < 0.05) (Fig. 7c). This illustrating that ZEB1 can be used as a prognostic marker for HCC.

## Discussion

LncRNAs can affect various biological processes through regulate the expression or activity of their downstream target genes, and are considered as one of the key regulatory factors for human cancer progression and drug resistance [5, 6]. There are increasing evidence suggest that lncRNAs present a vital role on the process of HCC occurrence and development and they are considered as a potential and novel biomarkers for the diagnosis and prognosis of HCC [22, 23]. Our present study screens out a novel lncRNA, lnc171, that increase remarkably in plasma exomes of HCC patients by RNA-sequencing and qRT-PCR. This lncRNA could badly impact HCC cells migration and invasion when it is knocking down, and this phenomenon has attracted our interest to study the mechanism of lnc171. LncRNA can combine with miRNAs, reducing the inhibitory regulation of miRNA on their target mRNAs’ translation [24]. Therefore, we used online software to predict the target miRNA—mir-873-5p of lnc171 and confirm their bonding.

mir-873-5p is one of the main members of mir-873 family that has been extensively studied. There are many genes targeted and regulated by mir-873-5p, these target genes have transcription regulation, catalytic activity, binding, and other molecular functions, which are closely related to the biological processes of cancer cells such as proliferation [25], apoptosis [26], migration [21], invasion [27]. mir-873-5p inhibit the invasion of papillary thyroid cancer cells by interfering with the expression of CXCL16 [28]. In addition, mir-873-5p could regulate triple-negative breast cancer cells’ proliferation and metastasis by inhibiting the MYC proto-oncogene, bHLH transcription factor [29]. The above research indicates that mir-873-5p was a tumor suppressor factor and has significant implications for the occurrence and development of cancer. Our study also demonstrated that mir-873-5p could significantly inhibited the migration and invasion of HCC cells.

In order to delve into the regulatory ceRNAs network that involved in lnc171 in HCC, we predicted the target genes that may be regulated downstream of mir-873-5p through prediction software, then found that ZEB1 may be regulated by mir873-5p. The regulatory relationship of between mir-873-5p and ZEB1 has also been reported in other studies [30, 31]. ZEB1 is a vital transcription factor that involved the procession in regulating epithelial-to-mesenchymal transition. And it has also been reported that ZEB1 was abnormally expressed in a variety of human cancers, which could promote cells’ migration, invasion, and metastasis [21, 27]. The study of Chen H shown that ZEB1 up-regulates the expression of centrosome protein 55 and affects the migration, invasion, and EMT of renal cell cancer cells [32]. Jesús MB confirmed that the role of ZEB1 silencing in reducing the migration and invasion ability of thyroid cancer cells [33]. In our study, it was found that lnc171 modulates increased both the mRNA or protein expression of ZEB1 by inhibiting mir-873-5p, thereby promoting the migration and invasion capacity of HCC cells. Therefore, we confirmed the lnc171—mir-873-5p—ZEB1 axis was an important pathway for regulating migration and invasion of HCC cells.

Alcohol abuse is one of the common risk factors for liver cancer [34]. Ethanol can induce liver damage and hepatic steatosis these lesions can cause alcoholic liver disease to progress to cirrhosis and eventually to HCC [35]. Ethanol has been proved to be a teratogenic agent and epigenetic regulator that regulates cell biological traits and functions by regulating the expression levels of multiple factors, leading to aggravating liver damage and accelerating the process of liver lesions [17–19]. In our study, we found that the stimulation of ethanol could increase the expression of lnc171 in HCC cells, and the migration and invasion ability of HCC cells was enhanced. In addition, ethanol stimulation reduced the expression of mir-873-5p and increased the expression of the ZEB1 protein which both downstream and regulated by lnc171. Therefore, we believe that ethanol can promote the migration and invasion of HCC cells through the lnc171—mir-873-5p—ZEB1 axis, making HCC malignant, which may be one of the mechanisms by which ethanol causes liver cancer. Inhibition of any factor of the lnc171—mir-873-5p—ZEB1 axis, especially the expression of lnc171, can inhibit the invasion and migration of HCC cells. Therefore, inhibitors targeting lnc171 may be one of the future treatment strategies of HCC. Promoting the expression of lnc171, such as alcohol intake, can promote the malignant progression HCC cells through the lnc171—mir-873-5p—ZEB1 axis. Therefore, reducing alcohol intake may be an important early warning measure for high-risk groups of HCC. Finally, we discussed the possibility that ZEB1 could be used as a biomarker for HCC and founding that ZEB1 is able to serve as a potential biomarker of HCC and helps to understanding the prognosis of HCC patients.

## Conclusions

In this study, we found that lnc171, a novel lncRNA, was significantly upregulated in HCC, which can competitively bind mir-873-5p and thus leads to less mir-873-5p-mediated ZEB1 inhibition, thereby enhancing the sensitivity of migration and invasion of HCC cells. We identified a ceRNA regulatory network, the lnc171—mir-873-5p—ZEB1 axis, in HCC. We also found that ethanol stimulation could up-regulated the increase of lnc171 in HCC cells, thereby enhance the migration and invasion of HCC cells through the ceRNA network in which lnc171 participated. Our study reveals that lnc171 plays an important role in HCC progression and highlights the potential of lnc171 as a therapeutic target for HCC.

### Electronic supplementary material

Below is the link to the electronic supplementary material.

Supplementary Material 1

Supplementary Material 2

Supplementary Material 3

Supplementary Material 4

## Data Availability

The datasets generated and/or analyzed during the current study are available at https://pan.baidu.com/s/1HAhfHlMuaeJnWToecUhYAA. The code is sydw.

## Abbreviations

HCC: Hepatocellular carcinoma
HC: Healthy control
lncRNA: Long noncoding RNA
ceRNA: Competing endogenous RNA
miRN A: microRNA
FBS: Fetal bovine serum
PS: Penicillin streptomycin
NC: Negative control
qRT-PCR: Quantitatice real time polymerase chain reaction
ZEB1: Zin-finger E-box binding homeobox
