# Supplementary figures and images for: Ethanol responsive lnc171 promotes migration and invasion of HCC cells via mir-873-5p/ZEB1 axis
Source: BMC Cancer. 2024 May 1;24:550. doi: 10.1186/s12885-024-12309-3 (PMC11064308; doi:10.1186/s12885-024-12309-3)

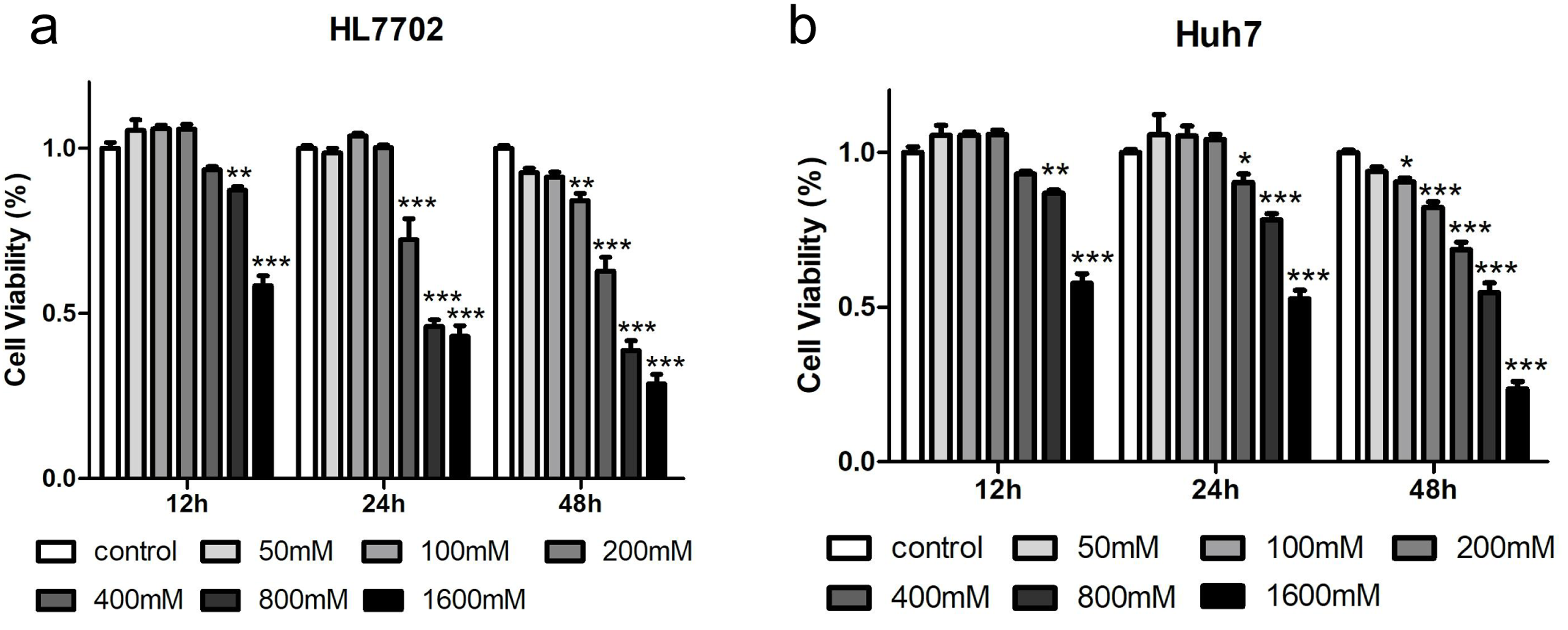

Supplement: Supplementary file 1 — Supplementary Material 1 [file 12885_2024_12309_MOESM1_ESM.tif]

GAPDH

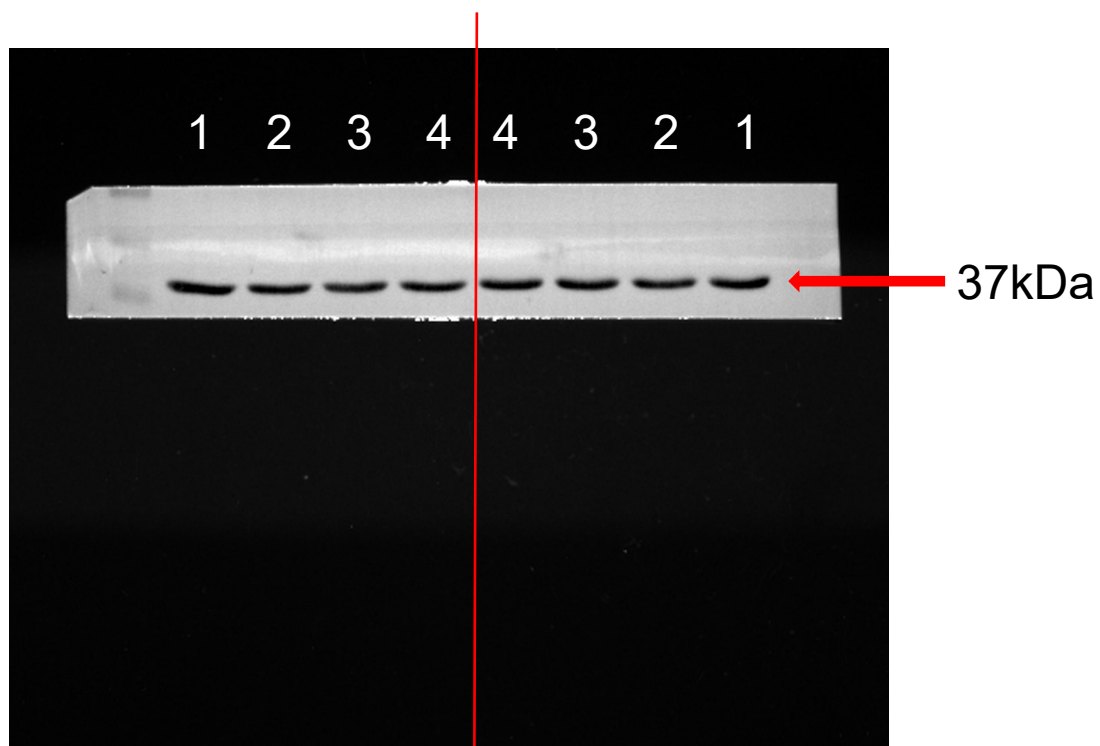

ZEB1

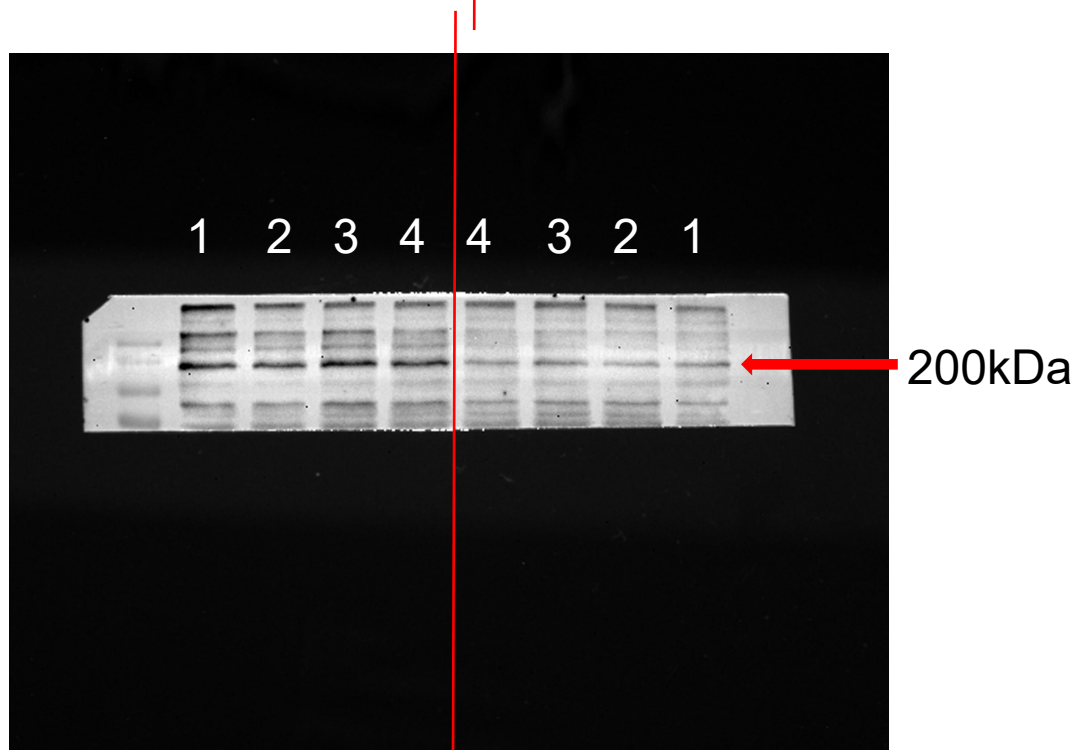

- 1:NC
- 2:si171
- 3:inhibitor
- 4:si171+inhibitor

Supplement: Supplementary file 4 — Supplementary Material 4 [file 12885_2024_12309_MOESM4_ESM.pdf]
