# Supplementary material for: Ethanol responsive lnc171 promotes migration and invasion of HCC cells via mir-873-5p/ZEB1 axis
Source: BMC Cancer. 2024 May 1;24:550. doi: 10.1186/s12885-024-12309-3 (PMC11064308; doi:10.1186/s12885-024-12309-3)
Supplement: Supplementary file 2 — Supplementary Material 2 [file 12885_2024_12309_MOESM2_ESM.docx]

Table S2 primer sequence

| Gene  name | primer sequence（5’~3’） | length（bp） |
| --- | --- | --- |
| lnc171 | F:ACAAGGAGCCAGAAGGGAAT | 20 |
|  | R:TTTCTTGGCTTGTGCTTGTG | 20 |
| ZEB1 | F:GGCATACACCTACTCAACTACGG | 23 |
| ACTB | R:TGGGCGGTGTAGAATCAGAGTC | 22 |
|  | F:CACCATTGGCAATGAGCGGTTC | 22 |
|  | R:AGGTCTTTGCGGATGTCCACGT | 22 |
| miR-873-5p | RT: GTCGTATCCAGTGCAGGGTCCGAGGTATT  CGCACTGGATACGACAGGAGA | 51 |
|  | F: GTATACGCAGGAACTTGTGAG | 21 |
|  | R: GTGCAGGGTCCGAGGT | 16 |
| U6 | RT: CGCTTCACGAATTTGCGTGTCAB | 23 |
|  | F: GCTTCGGCAGCACATATACTAAAAT | 25 |
|  | R: CGCTTCACGAATTTGCGTGTCAT | 23 |
