# Supplementary material for: Ethanol responsive lnc171 promotes migration and invasion of HCC cells via mir-873-5p/ZEB1 axis
Source: BMC Cancer. 2024 May 1;24:550. doi: 10.1186/s12885-024-12309-3 (PMC11064308; doi:10.1186/s12885-024-12309-3)
Supplement: Supplementary file 3 — Supplementary Material 3 [file 12885_2024_12309_MOESM3_ESM.docx]

Table S1 Sequence of siRNA and inhibitor and mimics of miR-873-5p

|  | sense(5’~3’) | antisense(5’~3’) |
| --- | --- | --- |
| si171-1 | GCUGUAGGCUACUGUUCAUTT | AUGAACAGUAGCCUACAGCTT |
| si171-2 | GCUACUGUUGUUCUACAAUTT | AUUGUAGAACAACAGUAGCTT |
| si171-3 | CCCAAAGGUUACCGAAGAATT | UUCUUCGGUAACCUUUGGGTT |
| siNC | UUCUCCGAACGUGUCACGUTT | ACGUGACACGUUCGGAGAATT |
| miR-873-5p inhibitor | (mA) (mG) (mG) (mA) (mG) (mA) (mC) (mU) (mC) (mA) (mC) (mA) (mA) (mG) (mU) (mU) (mC) (mC) (mU) (mG) (mC) | |
| miR-873-5p mimics | GCAGGAACUUGUGAGUCUCCU | GAGACUCACAAGUUCCUGCUU |
| mimics NC | UUGUACUACAAAAGUACUG | GUACUUUUGUGUAGUACAAUU |
